# Supplementary material for: Modeling glioblastoma heterogeneity as a dynamic network of cell states
Source: Mol Syst Biol. 2021 Sep 16;17(9):e10105. doi: 10.15252/msb.202010105 (PMC8444284; doi:10.15252/msb.202010105)
Supplement: Supplementary file 5 — Source Data for Figure 3 [file MSB-17-e10105-s001.zip › Figure3A_sourcedata/GSEA_3065/nosigdb_state2.GseaPreranked.1622718419215/FELDMAN_2020_S.html]

Details for gene set FELDMAN\_2020\_S[GSEA]

|  || Dataset | state2 |
| Phenotype | NoPhenotypeAvailable |
| Upregulated in class | na\_pos |
| GeneSet | FELDMAN\_2020\_S |
| Enrichment Score (ES) | 0.8352515 |
| Normalized Enrichment Score (NES) | 2.9322627 |
| Nominal p-value | 0.0 |
| FDR q-value | 0.0 |
| FWER p-Value | 0.0 |
Table: GSEA Results Summary

  

Fig 1: Enrichment plot: FELDMAN\_2020\_S      
 Profile of the Running ES Score & Positions of GeneSet Members on the Rank Ordered List

  

| PROBE | GENE SYMBOL | GENE\_TITLE | RANK IN GENE LIST | RANK METRIC SCORE | RUNNING ES | CORE ENRICHMENT || 1 | HMGB2 |  |  | 4 | 0.823 | 0.0179 | Yes |
| 2 | TYMS |  |  | 5 | 0.792 | 0.0355 | Yes |
| 3 | CENPU |  |  | 6 | 0.763 | 0.0524 | Yes |
| 4 | H2AFZ |  |  | 8 | 0.735 | 0.0687 | Yes |
| 5 | DHFR |  |  | 11 | 0.644 | 0.0828 | Yes |
| 6 | UBE2T |  |  | 12 | 0.643 | 0.0971 | Yes |
| 7 | MCM7 |  |  | 13 | 0.626 | 0.1110 | Yes |
| 8 | PCNA |  |  | 16 | 0.595 | 0.1240 | Yes |
| 9 | CDK1 |  |  | 18 | 0.577 | 0.1367 | Yes |
| 10 | MAD2L1 |  |  | 20 | 0.563 | 0.1491 | Yes |
| 11 | ATAD2 |  |  | 21 | 0.563 | 0.1616 | Yes |
| 12 | RRM2 |  |  | 22 | 0.556 | 0.1740 | Yes |
| 13 | DUT |  |  | 24 | 0.552 | 0.1862 | Yes |
| 14 | CLSPN |  |  | 27 | 0.536 | 0.1979 | Yes |
| 15 | ESCO2 |  |  | 30 | 0.530 | 0.2094 | Yes |
| 16 | DEK |  |  | 31 | 0.526 | 0.2211 | Yes |
| 17 | RPA3 |  |  | 36 | 0.511 | 0.2321 | Yes |
| 18 | GINS2 |  |  | 37 | 0.506 | 0.2433 | Yes |
| 19 | SMC2 |  |  | 39 | 0.493 | 0.2542 | Yes |
| 20 | ORC6 |  |  | 40 | 0.493 | 0.2651 | Yes |
| 21 | CENPK |  |  | 44 | 0.488 | 0.2756 | Yes |
| 22 | ZWINT |  |  | 45 | 0.488 | 0.2865 | Yes |
| 23 | ANP32B |  |  | 48 | 0.474 | 0.2968 | Yes |
| 24 | PTMA |  |  | 49 | 0.474 | 0.3074 | Yes |
| 25 | MCM3 |  |  | 51 | 0.464 | 0.3176 | Yes |
| 26 | RFC2 |  |  | 53 | 0.460 | 0.3277 | Yes |
| 27 | CENPM |  |  | 54 | 0.460 | 0.3379 | Yes |
| 28 | CHAF1A |  |  | 55 | 0.457 | 0.3481 | Yes |
| 29 | TK1 |  |  | 56 | 0.456 | 0.3582 | Yes |
| 30 | SIVA1 |  |  | 62 | 0.439 | 0.3674 | Yes |
| 31 | PTTG1 |  |  | 63 | 0.439 | 0.3772 | Yes |
| 32 | GMNN |  |  | 64 | 0.435 | 0.3868 | Yes |
| 33 | RANBP1 |  |  | 65 | 0.430 | 0.3964 | Yes |
| 34 | FBXO5 |  |  | 67 | 0.420 | 0.4056 | Yes |
| 35 | LMNB1 |  |  | 69 | 0.417 | 0.4148 | Yes |
| 36 | SNRPD1 |  |  | 74 | 0.408 | 0.4234 | Yes |
| 37 | ASF1B |  |  | 76 | 0.407 | 0.4323 | Yes |
| 38 | MCM4 |  |  | 79 | 0.399 | 0.4410 | Yes |
| 39 | MCM5 |  |  | 83 | 0.394 | 0.4494 | Yes |
| 40 | BRCA1 |  |  | 92 | 0.381 | 0.4570 | Yes |
| 41 | CDC45 |  |  | 95 | 0.377 | 0.4652 | Yes |
| 42 | RAN |  |  | 98 | 0.375 | 0.4733 | Yes |
| 43 | MCM10 |  |  | 100 | 0.373 | 0.4815 | Yes |
| 44 | GGCT |  |  | 102 | 0.373 | 0.4897 | Yes |
| 45 | DNAJC9 |  |  | 103 | 0.373 | 0.4980 | Yes |
| 46 | CDC6 |  |  | 106 | 0.372 | 0.5060 | Yes |
| 47 | FEN1 |  |  | 107 | 0.371 | 0.5143 | Yes |
| 48 | CACYBP |  |  | 109 | 0.366 | 0.5223 | Yes |
| 49 | SLBP |  |  | 112 | 0.357 | 0.5300 | Yes |
| 50 | HNRNPAB |  |  | 113 | 0.357 | 0.5379 | Yes |
| 51 | CENPV |  |  | 116 | 0.353 | 0.5456 | Yes |
| 52 | VRK1 |  |  | 118 | 0.350 | 0.5532 | Yes |
| 53 | POLD3 |  |  | 120 | 0.347 | 0.5608 | Yes |
| 54 | DNMT1 |  |  | 121 | 0.345 | 0.5685 | Yes |
| 55 | RFC4 |  |  | 126 | 0.339 | 0.5756 | Yes |
| 56 | CCDC34 |  |  | 127 | 0.339 | 0.5832 | Yes |
| 57 | SNRPB |  |  | 132 | 0.332 | 0.5901 | Yes |
| 58 | PA2G4 |  |  | 137 | 0.329 | 0.5970 | Yes |
| 59 | USP1 |  |  | 138 | 0.329 | 0.6043 | Yes |
| 60 | MIS18A |  |  | 142 | 0.322 | 0.6112 | Yes |
| 61 | NCL |  |  | 146 | 0.316 | 0.6179 | Yes |
| 62 | MCM6 |  |  | 149 | 0.311 | 0.6246 | Yes |
| 63 | SRSF7 |  |  | 155 | 0.306 | 0.6308 | Yes |
| 64 | NASP |  |  | 156 | 0.306 | 0.6376 | Yes |
| 65 | PSMC3IP |  |  | 159 | 0.305 | 0.6442 | Yes |
| 66 | CDCA3 |  |  | 160 | 0.304 | 0.6510 | Yes |
| 67 | HELLS |  |  | 161 | 0.304 | 0.6577 | Yes |
| 68 | CDCA7L |  |  | 167 | 0.301 | 0.6639 | Yes |
| 69 | DCTPP1 |  |  | 172 | 0.296 | 0.6700 | Yes |
| 70 | CARHSP1 |  |  | 178 | 0.294 | 0.6760 | Yes |
| 71 | ACAT2 |  |  | 180 | 0.294 | 0.6824 | Yes |
| 72 | CDCA4 |  |  | 184 | 0.292 | 0.6886 | Yes |
| 73 | CCNB2 |  |  | 190 | 0.286 | 0.6944 | Yes |
| 74 | FAM111B |  |  | 191 | 0.285 | 0.7008 | Yes |
| 75 | HSPA8 |  |  | 195 | 0.283 | 0.7067 | Yes |
| 76 | SMC3 |  |  | 197 | 0.283 | 0.7129 | Yes |
| 77 | MAGOHB |  |  | 199 | 0.282 | 0.7191 | Yes |
| 78 | HNRNPF |  |  | 205 | 0.279 | 0.7248 | Yes |
| 79 | TEX30 |  |  | 206 | 0.279 | 0.7310 | Yes |
| 80 | HSPB11 |  |  | 210 | 0.277 | 0.7368 | Yes |
| 81 | SRSF2 |  |  | 213 | 0.275 | 0.7427 | Yes |
| 82 | C19orf48 |  |  | 215 | 0.274 | 0.7487 | Yes |
| 83 | YWHAH |  |  | 221 | 0.271 | 0.7542 | Yes |
| 84 | ATAD5 |  |  | 222 | 0.269 | 0.7601 | Yes |
| 85 | EIF4EBP1 |  |  | 232 | 0.261 | 0.7650 | Yes |
| 86 | CENPA |  |  | 238 | 0.259 | 0.7702 | Yes |
| 87 | PGP |  |  | 244 | 0.254 | 0.7753 | Yes |
| 88 | MSH2 |  |  | 266 | 0.244 | 0.7784 | Yes |
| 89 | HAUS8 |  |  | 280 | 0.242 | 0.7824 | Yes |
| 90 | CCNE2 |  |  | 288 | 0.236 | 0.7869 | Yes |
| 91 | EXOSC9 |  |  | 298 | 0.233 | 0.7911 | Yes |
| 92 | POLR3K |  |  | 309 | 0.229 | 0.7951 | Yes |
| 93 | TMEM97 |  |  | 331 | 0.223 | 0.7978 | Yes |
| 94 | EIF1AX |  |  | 356 | 0.215 | 0.8000 | Yes |
| 95 | RPA2 |  |  | 367 | 0.212 | 0.8037 | Yes |
| 96 | DLGAP5 |  |  | 371 | 0.211 | 0.8080 | Yes |
| 97 | AURKA |  |  | 375 | 0.210 | 0.8124 | Yes |
| 98 | MSH6 |  |  | 380 | 0.209 | 0.8166 | Yes |
| 99 | HAT1 |  |  | 381 | 0.209 | 0.8212 | Yes |
| 100 | UNG |  |  | 398 | 0.200 | 0.8240 | Yes |
| 101 | BRCA2 |  |  | 427 | 0.193 | 0.8252 | Yes |
| 102 | LIMS1 |  |  | 436 | 0.193 | 0.8287 | Yes |
| 103 | EXOSC8 |  |  | 456 | 0.189 | 0.8308 | Yes |
| 104 | DERA |  |  | 471 | 0.185 | 0.8334 | Yes |
| 105 | CTNNAL1 |  |  | 532 | 0.176 | 0.8309 | Yes |
| 106 | RMI2 |  |  | 579 | 0.166 | 0.8296 | Yes |
| 107 | PPIF |  |  | 580 | 0.166 | 0.8333 | Yes |
| 108 | RFC3 |  |  | 617 | 0.161 | 0.8330 | Yes |
| 109 | MARCKS |  |  | 630 | 0.159 | 0.8353 | Yes |
| 110 | CCND1 |  |  | 840 | 0.134 | 0.8157 | No |
| 111 | TFDP1 |  |  | 874 | 0.130 | 0.8151 | No |
| 112 | EEF1E1 |  |  | 903 | 0.128 | 0.8149 | No |
| 113 | EIF2S1 |  |  | 1051 | 0.115 | 0.8016 | No |
| 114 | RPL22L1 |  |  | 1112 | 0.111 | 0.7976 | No |
| 115 | ARL6IP6 |  |  | 1134 | 0.109 | 0.7978 | No |
| 116 | TGIF1 |  |  | 1150 | 0.108 | 0.7986 | No |
| 117 | RNASEH2A |  |  | 1579 | 0.082 | 0.7544 | No |
| 118 | CDCA7 |  |  | 1684 | 0.078 | 0.7449 | No |
| 119 | ARL6IP1 |  |  | 2421 | 0.050 | 0.6668 | No |
| 120 | RAB13 |  |  | 3928 | 0.017 | 0.5050 | No |
| 121 | PAIP2 |  |  | 4690 | 0.004 | 0.4232 | No |
| 122 | CCNG1 |  |  | 4843 | 0.002 | 0.4069 | No |
| 123 | AKAP12 |  |  | 6471 | -0.025 | 0.2323 | No |
| 124 | EEF2 |  |  | 6630 | -0.028 | 0.2159 | No |
| 125 | EEF1A1 |  |  | 6690 | -0.029 | 0.2102 | No |
| 126 | BAMBI |  |  | 6859 | -0.033 | 0.1929 | No |
| 127 | PHLDA1 |  |  | 6867 | -0.033 | 0.1929 | No |
| 128 | LYPD1 |  |  | 6876 | -0.033 | 0.1927 | No |
| 129 | BEX4 |  |  | 6903 | -0.034 | 0.1907 | No |
| 130 | MANF |  |  | 7013 | -0.037 | 0.1798 | No |
| 131 | RPS27L |  |  | 7139 | -0.040 | 0.1672 | No |
| 132 | TPM1 |  |  | 7414 | -0.048 | 0.1388 | No |
| 133 | SLC20A1 |  |  | 7482 | -0.050 | 0.1327 | No |
| 134 | FTL |  |  | 7693 | -0.056 | 0.1113 | No |
| 135 | ZFAND6 |  |  | 7733 | -0.057 | 0.1084 | No |
| 136 | HES4 |  |  | 7819 | -0.060 | 0.1006 | No |
| 137 | YPEL5 |  |  | 7885 | -0.062 | 0.0950 | No |
| 138 | PLAU |  |  | 8131 | -0.073 | 0.0702 | No |
| 139 | BTG1 |  |  | 8156 | -0.075 | 0.0693 | No |
| 140 | MEST |  |  | 8534 | -0.099 | 0.0309 | No |
| 141 | DDIT4 |  |  | 8601 | -0.103 | 0.0261 | No |
| 142 | TAGLN |  |  | 8662 | -0.108 | 0.0221 | No |
| 143 | TSC22D1 |  |  | 8743 | -0.115 | 0.0160 | No |
| 144 | SOX4 |  |  | 9063 | -0.160 | -0.0148 | No |
| 145 | SCRG1 |  |  | 9200 | -0.191 | -0.0252 | No |
| 146 | SESN3 |  |  | 9266 | -0.219 | -0.0273 | No |
| 147 | SAT1 |  |  | 9279 | -0.224 | -0.0236 | No |
| 148 | NOVA1 |  |  | 9348 | -0.272 | -0.0249 | No |
| 149 | CALD1 |  |  | 9391 | -0.348 | -0.0217 | No |
| 150 | ARL6IP5 |  |  | 9420 | -0.467 | -0.0143 | No |
| 151 | MALAT1 |  |  | 9436 | -0.735 | 0.0004 | No |
Table: GSEA details [plain text format]

  

Fig 2: FELDMAN\_2020\_S: Random ES distribution      
 Gene set null distribution of ES for **FELDMAN\_2020\_S**
